# Case report: A unusual case of delayed propionic acidemia complicated with subdural hematoma

**DOI:** 10.3389/fneur.2022.1010636

**Published:** 2022-12-22

**Authors:** Zongzhi Jiang, Yuxin Fu, Xiaojing Wei, Ziyi Wang, Xuefan Yu

**Affiliations:** Department of Neurology and Neuroscience Center, The First Hospital of Jilin University, Changchun, China

**Keywords:** muscular pathology, late-onset, propionic acidemia, clinical exome sequencing, subdural hematoma

## Abstract

**Background:**

Propionic acidemia (PA) is an inherited autosomal recessive metabolic disorder that is classified as early-onset or late-onset, depending on the onset time of clinical symptoms. It clinically manifests as numerous lesions in the brain, pancreas, liver, and muscle. Muscle biopsies show myopathic changes, which help to distinguish late-onset propionic acidemia from other metabolic diseases involving muscles.

**Case presentation:**

A 19-year-old Chinese girl was admitted to the hospital because of poor eating and fatigue. Head magnetic resonance imaging suggested metabolic diseases, and we administered symptomatic support treatment. Her symptoms gradually worsened, and she began to show convulsions and disturbances of consciousness. Muscle pathology showed myopathy-like changes. The presence of organic acids in the blood and urine suggested PA. Genetic analyses identified two compound heterozygous mutations in the patient's PCCB gene, confirming the diagnosis of delayed PA.

**Conclusions:**

The muscle pathological examination of late-onset PA provides valuable information that is helpful for distinguishing delayed-onset PA from metabolic diseases. In the absence of a history of trauma, subdural hematoma may be a very rare complication of late-onset PA and can be regarded as a poor prognostic sign; therefore, it is suggested to perform head computed tomography as part of the routine neurological evaluation of PA patients.

## 1. Introduction

Propionic acidemia (PA) is an inherited autosomal recessive disease caused by the body accumulating propionic acid and its metabolites due to a deficiency in propionyl-CoA carboxylase (PCC) activity (1). PCC is a dodecameric mitochondrial enzyme that is composed of α and β subunits, which are encoded by genes PCCA and PCCB, respectively (2).

The clinical manifestations of the disease are complex and lack specificity. PA can be divided into early-onset and late-onset types, depending on the time that clinical symptoms manifest. The Apgar score of patients with early-onset PA is generally normal at birth and often changes within 3 months after birth (3). Delayed PA can occur from the age of 1 year to adulthood. Due to the different degrees of enzyme defects, the clinical manifestations of patients are heterogeneous (4). One method for investigating the pathogenesis of propionemia is through the accumulation of metabolites in the mitochondria, which results in mitochondrial dysfunction and leads to a series of neurological and muscle changes. Therefore, the pathological characteristics of myopathy caused by PA are similar to those of metabolic myopathy, which are characterized mainly by different sizes of muscle fibers, scattered distributions of vacuoles and cracks, and uneven enzyme activity in the cytoplasm. The ragged red fiber (RRF) and strong subdural hematoma (SDH)-reactive blood vessels (SSV) phenomena are evident in some patients (5).

We report a patient with PA screened by tandem mass spectrometry and urine gas chromatography–mass spectrometry. Two PCCB gene mutations were identified by clinical exome sequencing (CES), confirming a diagnosis of delayed PA. We also provide details on relatively rare pathological features of muscle tissue in PA.

## 2. Case presentation

**Figure 1:**
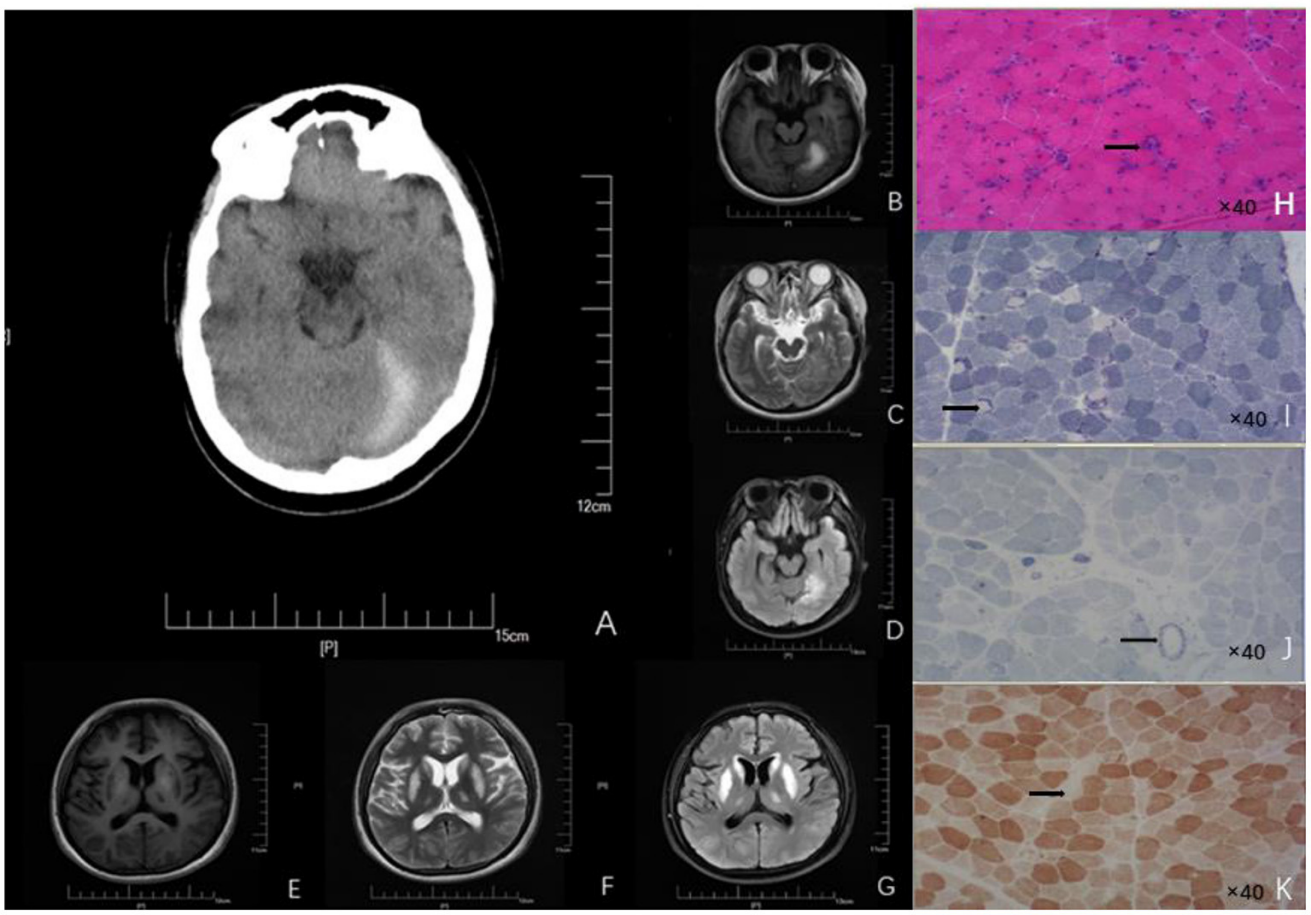
Neuroimages **(A–G)** and skeletal muscle biopsy **(H–K)** of this patient. **(A)** Craniocerebral CT showed high-density signal on the left side of the tentorial cerebellum, indicating subdural hematoma. Patchy abnormal signals are seen on the left tentorium of the cerebellum. **(B)** High intensity in T1 scan. **(C)** Slightly high intensity on T2 scan. **(D)** Significantly - high intensity on FLAIR scan). Symmetrical patchy abnormal signals could be found in bilateral lentiform nucleus, caudate nucleus head, and ventrolateral nucleus. **(E)** Low signal intensity in T1 scans. **(F)** Slight-higher signal intensity in T2 scans. **(G)** Obvious-high signal intensity in FLAIR scans. **(H)** HE findings of the skeletal muscle biopsy (right biceps muscle) revealed that the diameter of muscle fibers varied greatly; Atrophic muscle fibers were scattered in round and small circles together with large number of necrotic and regenerated muscle fibers (black arrow; 40× magnification). **(I)** NADH staining revealed uneven enzyme activity in some muscle fibers and dense staining around the muscle fibers (black arrow, 40× magnification). **(J)** SDH staining revealed SSV and several black arrow (black arrow; 40× magnification). **(K)** COX staining revealed negative-stained muscle fibers (black arrow; 40× magnification). HE, Hematoxylin-eosin; NADH, Nicotinamide adenine dinucleotide; CT, Computed tomography; FLAIR, Fluid attenuated inversion recovery.

**Figure 2:**
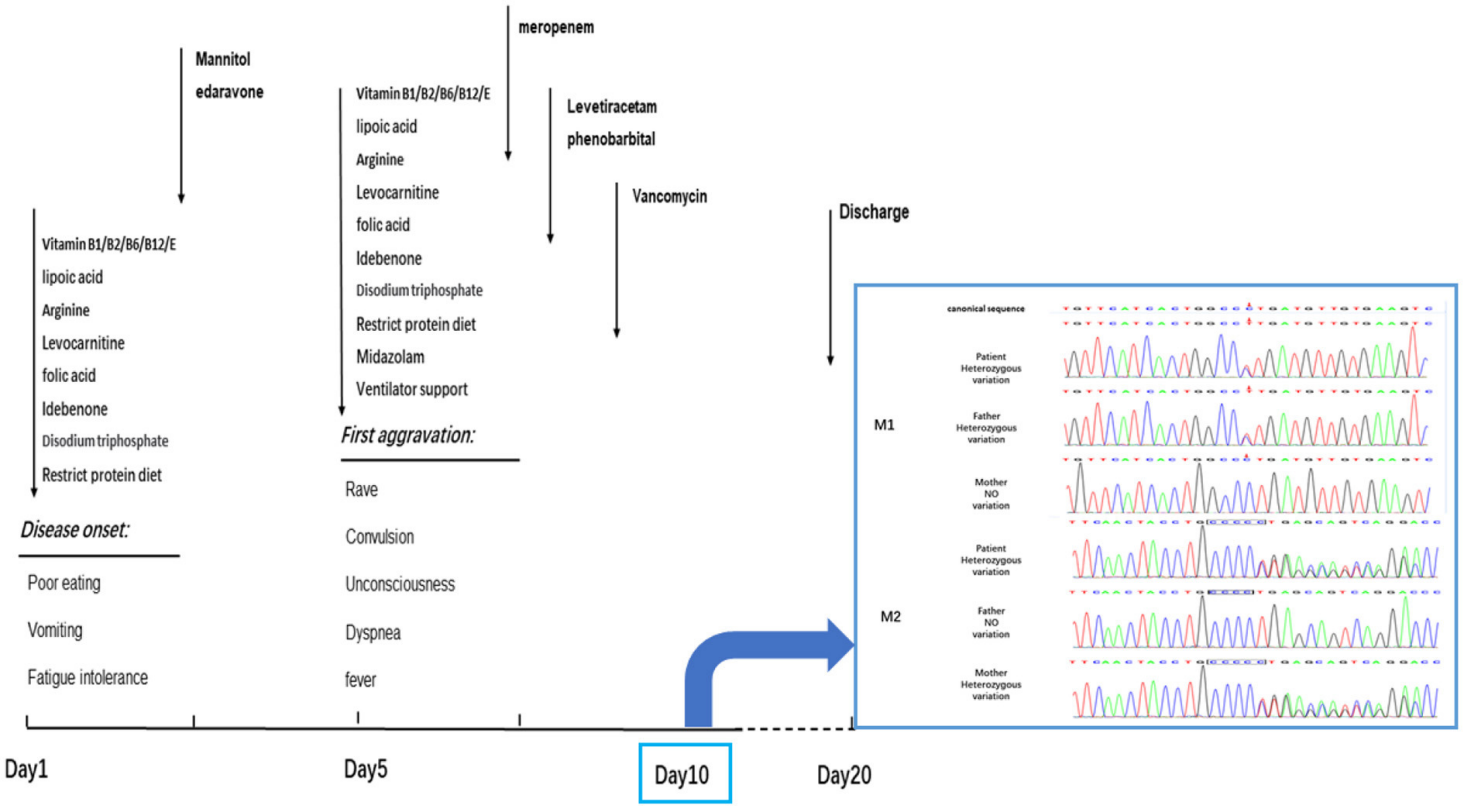
The progress of disease and the timeslot for the medical intervention. The patient got worsen on the 5th day during the hospitalization and then recovered slightly. Dark arrows represented the drug treatment. On the 10th day, the clinical exon sequencing (CES) showed one heterozygous missense mutation and one heterozygous frameshift mutation were detected by CES. M1, missense mutation; C.683C > T: P. Pro228Leu. M2, frameshift mutation; C.838–839insC: P. Leu280fsTer11. The results of Sanger sequencing revealed that the M1 mutations was inherited from her father, and M2 mutations from her mother. The patient was discharged on the 20th day without improving significantly.

A 19-year-old Chinese girl was admitted to our hospital because of poor eating, vomiting, and fatigue. She is the only child in the family, has normal growth and development, and is mainly vegetarian on weekdays. She denied smoking, drinking, food and drug poisoning, external injuries, and related family history. Examination of liver function showed elevated blood ammonia (108 μmol/L, normal range: 6–35 μmol/L). Brain magnetic resonance imaging (MRI) indicated symmetrical abnormal signals in bilateral basal ganglia, suggesting metabolic disease (Figure 1). Muscle pathological examination revealed myopathy-like changes (Figure 2) that anti-oxidative treatment, a restricted protein diet, and supplementation of coenzyme therapy did not improve.

The patient's clinical symptoms worsened 5 days after onset, including intermittent gibberish; convulsions began to appear that lasted for 1–2 min each time. The patient showed upper-limb flexion, lower-limb straightening, eye rolling, confusion, dyspnea, fever, and body temperature up to 40°C. She was treated with emergency endotracheal intubation and then transferred to the Neuroscience Intensive Care Unit. Neurological examination found that the patient was in a state of sedation, and her bilateral pupils were of equal diameter (about 3.0 mm in size) and sensitive to direct and indirect light. She had normal muscle tension in the extremities, positive bilateral pathological signs, no tendon reflex, was negative for a Kernig sign, and had no abnormal muscle tone. Gas analysis of the patient's blood indicated a pH of 7.440, carbon dioxide partial pressure of 28 mmHg, and oxygen partial pressure of 104.9 mmHg (4.39 mmol/L, normal range: 0.1–1.0 mmol/L). Blood analysis showed elevated white blood cells (17.24 x 10^9^/L, normal range: 3.5–9.5 x 10^9^/L), and elevated hypersensitive C-reactive protein (64.21 mg/L, normal range: 0–3.5 mg/L). A blood culture tested positive for Staphylococci, suggesting sepsis. The level of propionyl carnitine was 48.95 μM (normal range: 0.30–5.00 μM), and the ratio of propionyl carnitine to acetyl carnitine was 4.07 (normal range: 0.02–0.20). Urine organic acid analysis showed that 3-hydroxy propionic acid was 24.7 mol/mol creatinine (normal range: 0–4.0); methylmalonic acid was not detected in urine samples. EEG showed sharp–slow-wave and slow-wave activity in the left parietal–occipital region of high to extremely high amplitude. Head computed tomography (CT) showed symmetrical low densities and changes on the left side of the tentorial cerebellum (Figure 1), suggesting an SDH. Muscle pathological examination showed myopathy-like changes (Figure 2). The levels of ceruloplasmin, anticardiolipin antibody, serum amylase, serum lipase, 24-h free cortisol, tumor markers, infection markers, and renal function were not significantly abnormal. The biochemistry of cerebrospinal fluid from a lumbar puncture was in the normal range, the etiological test was negative, and the autoimmune encephalitis antibody in serum and cerebrospinal fluid were negative; thus, infectious diseases of the central nervous system were excluded. Based on these results, a diagnosis of PA was established.

Based on previous treatment, the patient was administered dehydration to reduce intracranial pressure and received targeted anti-infective and anti-epileptic treatments. The convulsions and fever symptoms were alleviated for a time; the hazy state of consciousness did not improve. The timeline of the aggravation and detailed treatment of symptoms is shown in Figure 1. After continuing symptomatic support treatment for 20 days, the patient did not respond to the treatment and their state of consciousness did not improve significantly. The family members asked that the patient to be discharged from the hospital and transferred to a specialist hospital for further treatment.

## 3. Clinical exome sequencing

Peripheral venous blood samples (3 mL) were collected from the patient and her parents. A sample of muscle tissue about 1.0 cm long and 0.5 cm wide was taken from the patient. A genomic DNA extraction kit (TIANGEN, China) was used to extract DNA from the blood samples, and a mitochondrial DNA extraction kit (TIANGEN, China) was used to obtain mitochondrial DNA from the muscle tissue. The quantity/quality of the nuclear DNA and mitochondrial DNA were evaluated using a ONE DROP OD-1000 spectrophotometer (Black Horse Medical Instruments Co., LTD, China) and agarose gel electrophoresis. The DNA samples were delivered to Beijing Zhiyin Oriental Medical Inspection Co., Ltd for CES analysis. Two compound heterozygous mutations were detected by Sanger sequencing. No variation sites with high pathogenicity were found in the mitochondrial DNA sequencing analysis.

The results of CES showed that: (1) the mutations in the PCCB gene, c.838 (exon 8) and c.839 (exon 8), were frameshift mutations, which are expected to cause a coding disorder of the PCCB protein at the position-280 isoleucine. In addition, it terminates translation after the coding of 11 amino acids, forming a truncated protein, resulting in the loss of the protein function. (2) This was a missense mutation, which leads to the transformation of codon 228 of the PCCB gene from proline to isoleucine. According to the American College of Medical Genetics and Genomics (ACMG) criteria and guidelines for explaining sequence variation, the frequencies of these two mutations in all normal-population databases are <0.0005, and both are compound heterozygous mutations. The results of Sanger sequencing confirmed that the mutations M1 and M2 were inherited from the mother and father, respectively (Figure 2). These results confirm that the patient's healthy parents are heterozygous carriers. The mutation in the PCCB gene can lead to autosomal recessive hereditary PA.

## 4. Discussion

PCC is located in the mitochondria and can catalyze the conversion of propionyl-CoA to methylmalonyl-CoA. Mutation of PCCA or PCCB can lead to the loss of PCC activity, cause the accumulation of propionyl-CoA in the body, and produce a high concentration of intermediate metabolites (e.g., propionic acid, 3-hydroxypropionic acid, and methyl citrate) by activating an accessory pathway. This causes circulatory, neurological, endocrine, and other systems to also be impaired (3, 5).

The clinical manifestations of this patient during the course of the disease were a series of neurological symptoms that may be the acute manifestations of delayed PA, including gibberish, convulsions, and disturbance of consciousness. Although the neuropathophysiology of PA is not fully understood, studies have found that excessive accumulation of some metabolites exceeds the neurotoxicity caused by physiological concentrations (e.g., propionic acid, glycine, and blood ammonia) and long-term fat decomposition disorders, leading to over-reliance on the carbohydrate energy supply (6). As a result, the lack of neuronal energy storage and oxidative damage can have acute or chronic effects on energy metabolism. Glial cell expression and the brain's physical structure among neurons can be impaired synchronously. Therefore, neurological impairment is more common in patients with late-onset PA than in patients with early-onset PA.

In addition to assessing nervous system symptoms, evaluating neuroimaging changes are also important for the diagnosis of delayed PA (7). The common neuroimaging features of delayed PA are symmetrical basal ganglia lesions, especially lesions of the globus pallidus, different degrees of cistern, sulcus widening, delayed myelination, dysplasia of the corpus callosum, and supratentorial white matter edema (8). In this case, MRI resulted in partial findings; the imaging features were patchy abnormal signals in the bilateral basal ganglia. The involvement site in this patient was consistent with a common injury site of late-onset PA. Although the specific pathogenesis is not clear, a reasonable explanation is that the level of energy metabolism in areas such as basal ganglia and cerebral foot is higher; therefore, it is more vulnerable to damage than other regions (9).

The accumulation of organic acids and hyperammonemia aggravate neuronal damage through neurotoxicity. In addition to the common neuroimaging changes, there are also other special imaging findings. For example, Velasco-Sánchez et al. reported a PA patient with cerebellar hemorrhage (10). Head CT of this patient showed an SDH on the left tentorium of the cerebellum, but there was no history of trauma, which is very rare in patients with delayed PA. There are two main reasons to be considered for this. (1) Vascular endothelial cell injury: the loss of PCC activity leads to the accumulation of propionyl-CoA in the mitochondria, decreasing mitochondrial ATP production and increasing mitochondrial Ca^2+^ consumption, which leads to the activation of cell-membrane Ca^2+^-exchange proteins and an intracellular calcium overload; this promotes the activation of phospholipases, proteases, and nucleases, resulting in vascular endothelial cell injury and shedding, ultimately resulting in increased vascular wall permeability and the formation of an SDH. (2) Vascular endothelial cell contraction due to the accumulation of high concentrations of organic acids in patients' cells stimulates an inflammatory reaction, leading to the recruitment of histamine, bradykinin, leukotriene, and other inflammatory media, and infiltration. Endothelial cell contraction would also result in increased vascular permeability.

We suggest that relying only on the abnormal changes evident on MRI and CT is not sufficient to distinguish PA from other metabolic diseases, especially Leigh syndrome. Therefore, muscle pathological examination should be performed to detect the presence of other metabolic diseases. In muscle pathology, Leigh syndrome usually shows different sizes of muscle fibers, in which lipid deposition is common, atrophic muscle fibers are mostly type II, atypical RRFs can be seen, and loss of COX enzyme activity is evident in some patients' muscle fibers and blood vessel walls (11). In contrast, the muscle pathology in mitochondrial encephalomyopathy with lactic acidosis and stroke-like seizure syndrome (MELAS) tends to show a scattered distribution of RRFs and strong SSVs (12). In our case, great variation in the muscle fiber diameter was observed, and a large number of necrotic and regenerated muscle fibers were evident. RRF was not found in GT staining. NADH staining showed that some of the enzyme activities were uneven. SSV was evident on SDH staining. Several COX-negative muscle fibers were made visible by COX staining. Several necrotic muscle fibers were positive for acid staining. Staining for NADH, COX, and SDH showed that the enzyme activity in some muscle fibers was uneven, indicating that the mitochondrial inner membrane respiratory chain function was abnormal. Combined with previous studies, this suggested that a decrease in PCC activity caused an accumulation of propionyl CoA in the mitochondria (13). This would inhibit the electron transfer function of complex IV cytochrome C oxidase in the mitochondrial inner membrane and interrupt the electron transfer process of the mitochondrial respiratory chain (14). Abnormal oxidative phosphorylation involves skeletal muscle, making the pathological characteristics of muscle in PA similar to that of metabolic myopathy. This suggests that PA involving skeletal muscle should be classified into the category of metabolic myopathy. The pathological features of delayed-onset PA in muscles are reported rarely in the literature. Therefore, our case provides valuable information about the pathological features of muscles in PA.

In conclusion, our unusual case provides valuable information on the pathological muscle features of PA, having implications for future diagnoses and treatments. Because muscle pathological examination of delayed PA is very rare, this case provides valuable information on the pathological features of PA muscles. It is clear that, in the absence of a history of trauma, SDH may be a rare complication of delayed PA. To prevent the disease from aggravating due to cerebrovascular complications, it is advisable to perform a head CT examination as part of the routine neurological evaluation of PA patients. Early detection is very important for starting physiotherapy and nutritional diet intervention as soon as possible; these can reduce the disturbance of growth and development caused by a metabolically decompensated environment.

## Data availability statement

The original contributions presented in the study are included in the article/supplementary material, further inquiries can be directed to the corresponding author/s.

## Ethics statement

Written informed consent was obtained from the individual(s) for the publication of any potentially identifiable images or data included in this article.

## Author contributions

ZJ and YF wrote the manuscript. XW and ZW examined and treated the patient. XY participated in revising the manuscript. All authors contributed to the article and approved the submitted version.
